# Biological and genomic characteristics of chicken-derived *Riemerella anatipestifer* in China

**DOI:** 10.3389/fmicb.2025.1652106

**Published:** 2025-08-29

**Authors:** Yuxia Zhang, Xiaoli Wang, Yongming Wang, Jiazhi Sun, Wenwen Dong, Kai Meng, Guiming Li, Xiaoyuan Yuan

**Affiliations:** ^1^Poultry Institute, Shandong Academy of Agricultural Sciences, Jinan, China; ^2^Shandong Provincial Key Laboratory of Livestock and Poultry Breeding (PKL2024B15), Jinan, China; ^3^Binzhou Polytechnic, Binzhou, China; ^4^Shandong Huahong Biological Engineering Co., Ltd., Binzhou, China

**Keywords:** *Riemerella anatipestifer*, chicken, isolation and identification, pathogenicity, genome

## Abstract

**Introduction:**

*Riemerella anatipestifer* (*R. anatipestifer*) is a significant bacterial pathogen responsible for serositis, perihepatitis, and encephalitis in waterfowl. Although infections have been extensively reported in ducks, geese, and turkeys, cases in chickens are rarely documented. However, recent evidence indicates that it has emerged as a growing threat to chickens in China in recent years.

**Methods:**

In this study, we collected 120 clinical samples from 30 chicken farms across Shandong and Henan provinces (2023 to 2024) and conducted a comprehensive etiological investigation involving bacterial isolation, antibiotic susceptibility test and genomics analysis. The pathogenic of two *R. anatipestifer* strains (JN01 and BZ), belonging to serotypes 1 and 10, was evaluated.

**Results:**

Our findings revealed *R. anatipestifer* as the primary causative agent of the outbreak, with 28 strains successfully isolated. Serotypes 1 and 10 were identified as the predominant epidemic types, while serotypes 7 and 6 appeared sporadically. All isolates exhibited varying degrees of resistance to 15 commonly used antibiotics. Pathogenicity assessment via chicken embryo lethality assay demonstrated that JN01 strain displayed lower virulence than the BZ strain. Experimental infection of specific-pathogen-free (SPF) chickens with a challenge dose of 1 × 10^8^ CFU per chicken successfully reproduced clinical symptoms, with high bacterial loads detected in joint cavities and brains at 10 days post-inoculation. The complete genome of the isolated JN01 was 2,284,590 bp, as determined by third-generation sequencing. Phylogenetic analysis of the whole-genome sequence showed that JN01 is most closely related to strains isolated from ducks within the same clade. Notably, strains from different hosts, including chicken, duck, goose, and tadorna, did not form distinct independent branches but were intermixed throughout the evolutionary tree.

**Discussion:**

Our findings demonstrated *R. anatipestifer* exhibits remarkable host adaptability to chickens. Both the age-dependent susceptibility and clinical manifestations in boilers are essentially consistent with those observed in ducks. The serotypes prevalent in chicken flocks share both similarities and differences with those in duck flocks. Notably, its lethality to chickens is significantly lower than that to ducks. These findings enhance our understanding of the epidemiology and pathogenicity of *R. anatipestifer* from chicken, providing a scientific foundation for targeted disease prevention and control measures.

## Introduction

The Gram-negative *R. anatipestifer* belongs to the Flavobacteriaceae family and causes acute septicemia and exudative diseases in ducklings mainly aged 1–8 weeks, leading to high morbidity and mortality (Yehia et al., 2023; Liu et al., 2024). This pathogen is globally distributed, causing severe economic losses in the duck farming industry. To date, 25 serotypes of *R. anatipestifer* have been identified, with notable variations in virulence (Cheng et al., 2003). Weak cross-protective immunity among different serotypes poses challenges for vaccine immunity (Kang et al., 2018).

Historically, *R. anatipestifer* infections were predominantly associated with waterfowl (Surya et al., 2016). However, since late 2022, outbreaks in broiler and layer flocks have emerged across China, causing substantial economic losses (Zhang et al., 2025). In layers, *R. anatipestifer* infection leads to reduced egg production, decreased hatching rates, and the occurrence of “jelly-like” dead embryos (Chen et al., 2024). However, the losses caused by *R. anatipestifer* infection in broilers should not be underestimated either, as it has shown rapid geographical spread and a sharp increase in isolation rates. *R. anatipestifer* mainly affects 25-to 35-day-old broilers, causing clinical signs such as listlessness, sneezing and torticollis, often progressing to neurological symptoms like leg paralysis and tarsal joint swelling. Notably, abnormal deaths in most affected flocks persist until the slaughter period, with a cumulative mortality rate ranging from 2% to 10%. A key pathological feature is fibrinous exudate covering the liver, heart, and air sacs.

At present, antibiotic treatment remains the primary control measure for *R. anatipestifer* infections. However, studies indicate this bacterium has natural resistance to multiple antibiotics (Quan et al., 2023; Dong et al., 2024). Moreover, existing vaccines offer limited protection for chicken due to differences between serotypes and circulating strains, as well as host-specific differences.

Given these challenges, this study focuses on the infection of *R. anatipestifer* in chickens, with a focus on prevalence situation, biological characteristics and genomic features of circulating strains. These findings aim to provide insights for developing targeted prevention and control strategies against this emerging poultry pathogen.

## Materials and methods

### Sample collection and suspected pathogen detection

In this study, 120 cases of chicken tissue samples (including liver, brain) and joint fluid were collected from 30 chicken farms (including 15 broiler farms and 15 layer farms) where chickens exhibiting serositis, neurological symptoms or blocked fallopian tubes in Shandong and Henan provinces in China between 2023 and 2024. All fresh tissues were inoculated onto MacConkey agar and tryptone soya agar (TSA) supplemented with 5% calf serum, followed by incubation at 37 °C under 5% CO_2_ for 24–48 h.

In response to respiratory symptoms observed in clinical chicken flocks, we conducted pathogen screening targeting key etiological agents such as avian influenza virus, Newcastle disease virus and Infectious bronchitis virus. Concurrently, considering the manifestations of hepatitis and joint swelling, tests for adenovirus and reovirus infections were also carried out. Additionally, Egg Drop Syndrome virus and Avian encephalomyelitis virus were detected for the presence of clinically reduced egg production in layers and neurological symptoms, respectively. To rule out viral co-infections, PCR/RT-PCR assays were performed for potential pathogens, using published primers (Channa et al., 2021; Dimitrov et al., 2019; Ma et al., 2019; Yuan et al., 2021; Roussan et al., 2012; Cha et al., 2013; Goto et al., 2019). All primers were synthesized by BGI Genomics Institute. Viral RNA/DNA were extracted from tissue supernatants using a commercial kit (Tiangen, China), and PCR/RT-PCR (TakaRa, China) were performed according to standard protocols.

### Isolation, identification and serotyping

Purified bacterial colonies were subjected to Gram staining (Solarbio, China) and the morphologies were observed under a light microscope. Serotyping was performed using slide agglutination with polyclonal antisera (Zhang et al., 2024). Genomic DNA was extracted using a bacterial DNA kit (Tiangen, China), and species identification was confirmed by PCR of 16S rDNA (primers: F-5′AGAGTTTGATCMTGGCTCAG3′, R-5′AAGGAGGTGATCCAGCC3′, predicted size: 1,465 bp) and *R. anatipestifer*-specific PCR (primers: F- 5′TTA GATAGTTGGTGAGGTAA3′, R- 5′ATCGGTGTTCTGAGTA AT3′, predicted size: 475 bp) (Chen et al., 2010).

### Antibiotic sensitivity test

Antibiotic susceptibility testing was performed using the Kirby–Bauer disk diffusion method according to the Clinical and Laboratory Standards Institute (CLSI M100) guidelines (Clinical and Laboratory Standards Institute, 2023). The cultures of isolates were adjusted to 0.5 McFarland turbidity and spread onto Mueller–Hinton agar. Then the disks (Hangzhou Microbial Reagent Co., Ltd., Hangzhou, China) were placed on the agar surface, with *E. coli* ATCC25922 as a quality control. A panel of 15 antibiotics disk represent different classes were tested, including β-lactams (amoxicillin, cephalexin, cefoperazone sodium), Aminoglycosides (Gentamicin, Streptomycin, Amikacin, Neomycin, Spectinomycin), Fluoroquinolones (Enrofloxacin), and others (Florfenicol, Tetracycline, Polymyxin B, Fosfomycin, Lincomycin). Inhibition zone diameters were measured in millimeters after 24 h incubation at 37 °C, and inhibition results were interpreted according to CLSI criteria (Clinical and Laboratory Standards Institute, 2023).

### Pathogenicity in SPF chicken embryo and chicken

Chicken embryos were used to determine the virulence of *R. anatipestifer*, as previously described (Seo et al., 2013). One hundred 10-day-old SPF chicken embryos were divided into 10 groups (*n* = 10) and inoculated via the allantoic cavity with JN01 (serotype 1) or BZ strains (serotype 10) at concentrations of 1 × 10^8^, 1 × 10^6^, 1 × 10^4^, 1 × 10^2^ CFU, or PBS, respectively. Embryo survival was monitored every 12 h for 5 days.

Meanwhile, thirty 30-day-old SPF chickens (reference the age of onset) were randomly divided into 3 groups (*n* = 10). Two groups received injections of 300 μL JN01 or BZ strain, respectively, at the same concentration (1 × 10^8^ CFU) via the intramuscular route, while the control group received an equal volume of PBS. Each group was separately isolated and raised. Clinical symptoms, morbidity, and mortality were recorded for 10 days (based on the preliminary experiment). Cloacal swabs were collected at 0, 2, 4, 6, 8, and 10 dpi for qPCR quantification of bacterial DNA. At 10 dpi, tissues (brain, liver, heart) and joint fluid were collected for pathological sections and bacterial load analysis via qPCR. A SYBR Green-based qPCR assay was performed with specific primers designed targeting the conservative sequence of 16S rDNA gene. RA-F:5′-GCT GGA ATG AGT AGT GTA G-3′, and RA-R: 5′-GCT TAG TCT CTG AAC CAT ATA G-3′, targeting 190 bp. The thermal profile of qPCR included an initial denaturation step at 95 °C for 30 s, followed by 40 cycles of denaturation at 95 °C for 5 s, annealing and extension at 60 °C for 30 s, during which fluorescence data were acquired. Meanwhile, the *R. anatipestifer* of duck-origin identified and preserved in our laboratory was used as the positive control, and ddH2O was used as the negative control.

### Ethics statements

The animal-use protocols were approved by the Animal Experiment Ethics Committee of Shandong Academy of Agricultural Sciences.

### Genomic and phylogenetic analysis

The genomic DNA of strain JN01 was extracted using a Roche kit (Roche, Switzerland) and quality-assessed via DeNovix DS-11 (Liu et al., 2019). Libraries were constructed using PacBio and Oxford Nanopore technologies, and the third-generation sequencing was performed on Illumina NovaSeq and Nanopore platforms. Genomic assembly and correction were carried out using Pilon v1.24 and Hifiasm v0.19.5. Functional annotation was performed via NCBI PGAAP, KEGG, GO, CARD, and CAZy databases. Phylogenetic analysis of the whole-genome was conducted using IQ-TREE2 (TVM+F+ASC+R4 model) with 1,000 bootstrap replicates, incorporating the isolated strain JN01 and the representative reference strains from different hosts, different eras and different countries.

## Results

### Isolation, identification and serotyping

A total of 28 *R. anatipestifer* strains were successfully identified by isolation culture and PCR from 120 cases with neurological symptoms and serositis. The highest detection rate was obtained from livers covering a fibrinous exudate, followed by joint fluid with tarsal joint swelling in broilers. According to the statistics of pathogen isolation results, the isolation success rate of *R. anatipestifer* in broilers (33.3%) is higher than that in layers (13.3%), and among broilers, white-feathered broilers account for a relatively higher proportion.

**FIGURE 1:**
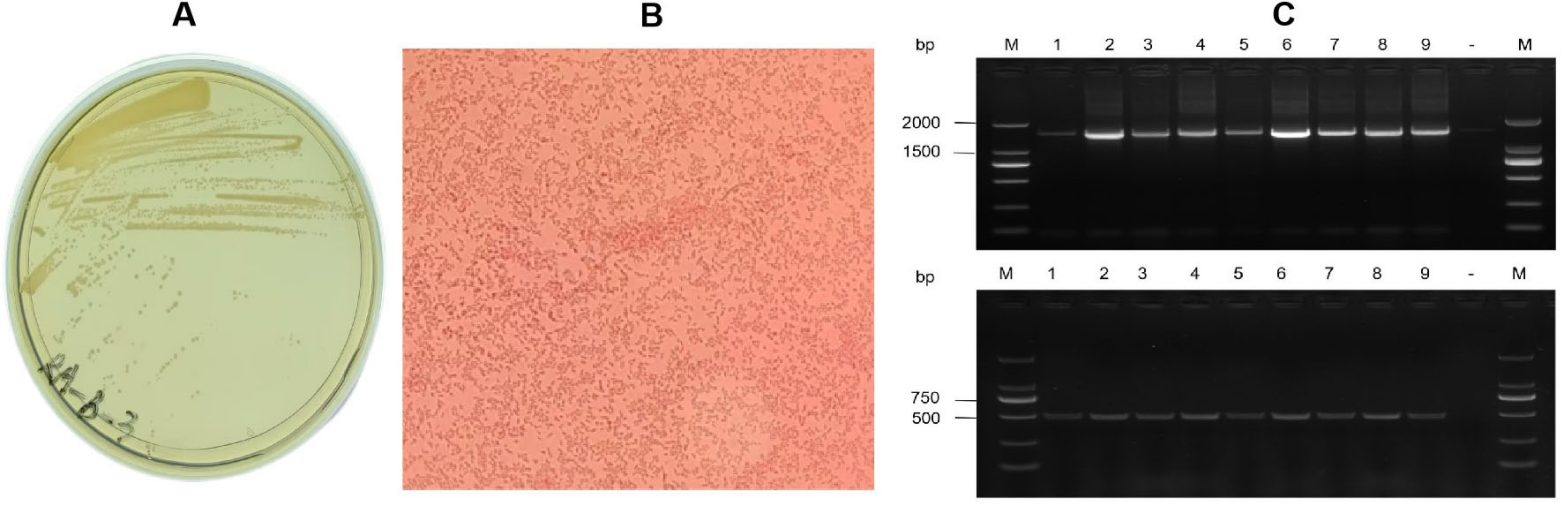
Isolation and identification of *Riemerella anatipestifer* JN01 strain. **(A)** Growth of the JN01 strain on TSA agar containing 5% FBS at 37 °C for 48 h with 5% CO_2_. **(B)** Gram staining of the JN01 strain (1000× magnification). **(C)** Agarose gel electrophoresis. Lane M: DNA marker (2000); Lane1-9 represents the positive sample; “-” is the negative sample.

On TSA agar, *R. anatipestifer* formed round, smooth grayish-white colonies with a diameter of about 1–2 mm (Figure 1A), single or a few arranged without spores (Figure 1B). PCR assays using 16S rDNA and species-specific primers yielded amplicons of expected sizes (1,465 bp and 475 bp, respectively) (Figure 1C). Among 28 strains, 16 strains (57.1%) were serotype 1, 6 strains (21.4%) as serotype 10, 3 strains as serotype 7 and 1 strain as serotype 6; two strains remained untyped. In which, 3 cases co-infected with *E. coli*, and 2 cases with H9 AIV were found. Serotype 1 and serotype 10 were dominantly associated with broiler and layer flocks, respectively.

### Antibiotic sensitivity test

**FIGURE 2:**
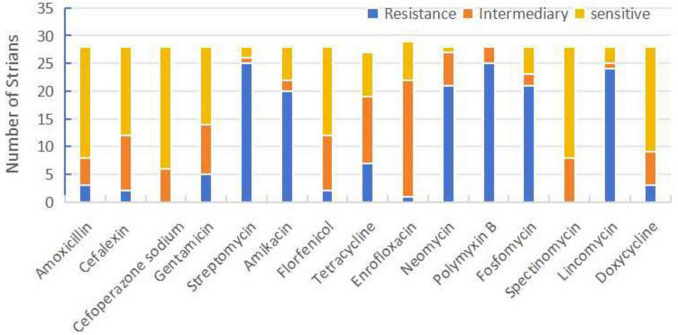
Sensitivity, moderate sensitivity, and resistance ratios of *Riemerella anatipestifer* isolates to 15 antibiotics.

All isolates exhibited different degrees of drug resistance for 15 common antibiotics in veterinary clinical practice, with sensitivity to cefoperazone sodium, florfenicol, doxycycline, and spectinomycin; while streptomycin, amikacin, polymyxin B, and phosphomycin were found to be resistant in most of the isolated strains (Figure 2).

### Pathogenicity detection

**FIGURE 3:**
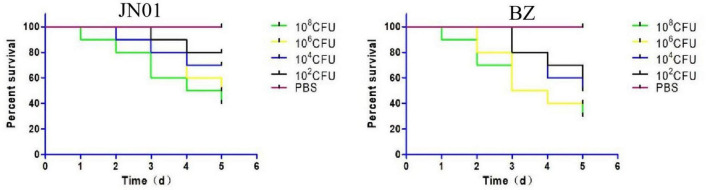
The survival rate of chicken embryos infected by JN01 strain (left) and BZ strain (right).

When SPF chicken embryos were inoculated with 10^2^ to 10^8^ CFU into the allantoic cavity, JN01 and BZ strains both exhibited dose-dependent lethality. At 1 × 10^8^ CFU, BZ caused 70% mortality within 5 days, significantly higher than JN01 (60%). Lower doses prolonged the survival times of inoculated chickens, with no mortality in the PBS group (Figure 3).

**FIGURE 4:**
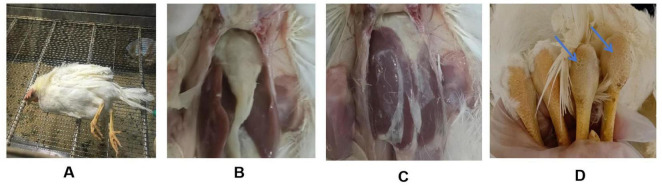
Clinical manifestations of SPF chickens challenged with the *Riemerella anatipestifer* isolates. **(A)** A paralyzed chicken lying on its side; **(B,C)** Fibrinous exudate on the surface of the chicken’s heart and liver; **(D)** Swelling of the tarsal joint in the challenged chicken (right) compared to the control group (left).

The pathogenicity of the two isolates was further evaluated in SPF chickens. Lethargy and anorexia were observed in inoculated SPF chickens at 1 dpi. Subsequently, the infected chicken exhibited ataxia and increased secretion of ocular and nasal fluids. Death was found two days later in BZ group. JN01-inoculated chickens showed leg swelling (40%), while BZ-inoculated chickens exhibited earlier neurological symptoms (tremors, ataxia) and higher morbidity (70% leg swelling, 10% mortality). The most notable pathological change was the presence of fibrinous serositis in both groups of inoculated chickens, with extensive exudate in the heart and liver (Figure 4).

**FIGURE 5:**
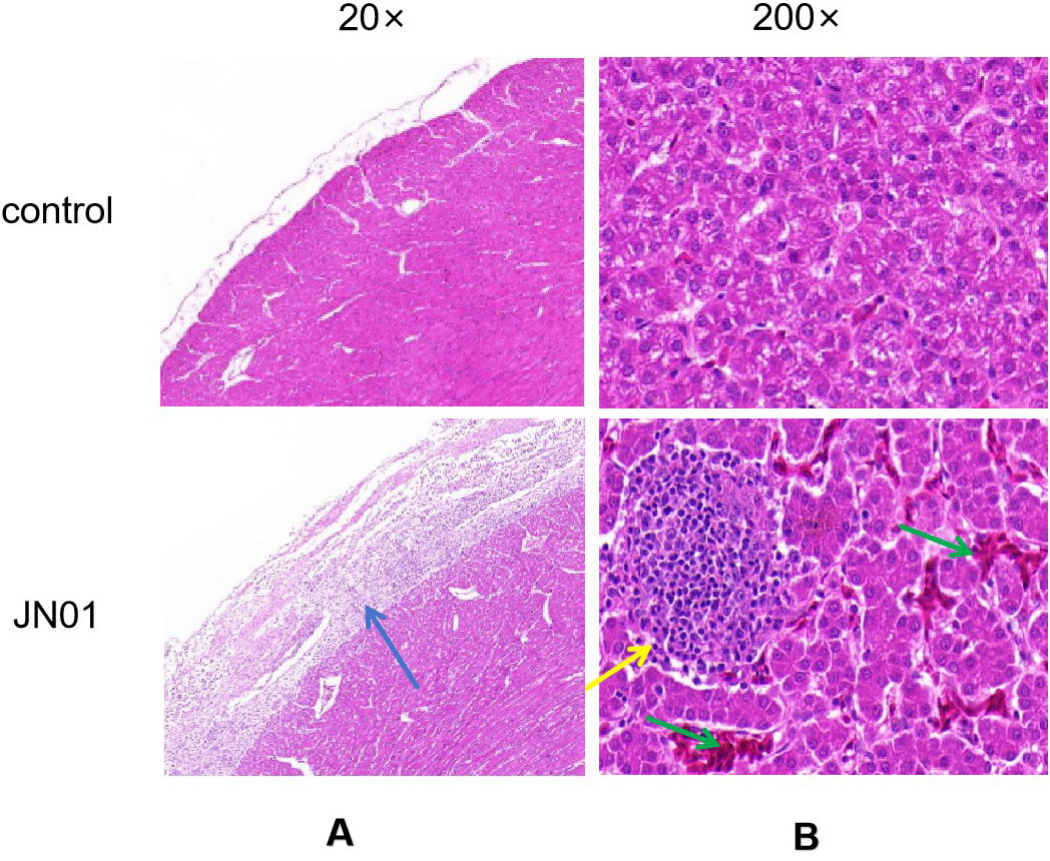
Histopathologic analysis of organ tissue from chickens. **(A)** Heart: significant thickening of the pericardium, and increased interstitial space were observed, with a large amount of inflammatory cells exuding from the epicardium; **(B)** Liver: inflammatory cells infiltrated the liver tissue; as indicated by the green arrow, hemorrhage in the liver tissue.

**FIGURE 6:**
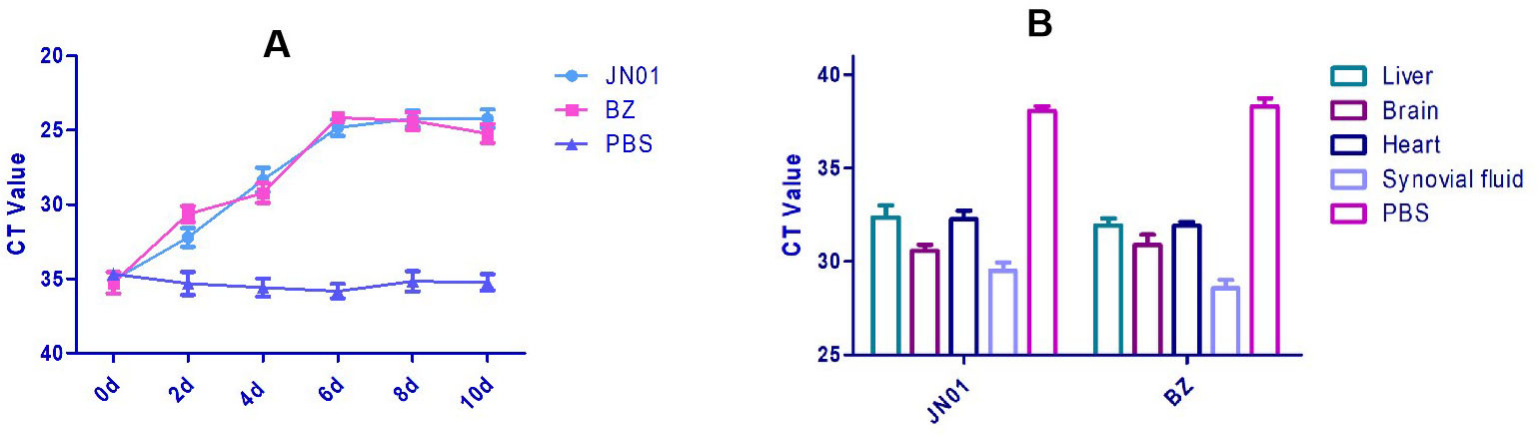
**(A)** Quantitation detection of the DNA of *Riemerella anatipestifer* in cloacal swabs collected from SPF chickens after artificial challenge by qPCR method. **(B)** Quantitative detection of the DNA of *Riemerella anatipestifer* in different tissues using the qPCR method after artificial challenge.

Significant thickening of the pericardium and widening of interstitial spaces were observed in heart. Inflammatory cell infiltration was seen in the liver tissue (Figure 5). qPCR detected bacterial DNA in cloacal swabs as early as 2 dpi, peaking at 6 dpi, with no significant difference between different strains (Figure 6A). Tissue analysis showed the highest bacterial loads in joint fluid and brain at as the 10 dpi (Figure 6B).

### Genomic and phylogenetic analysis

**FIGURE 7:**
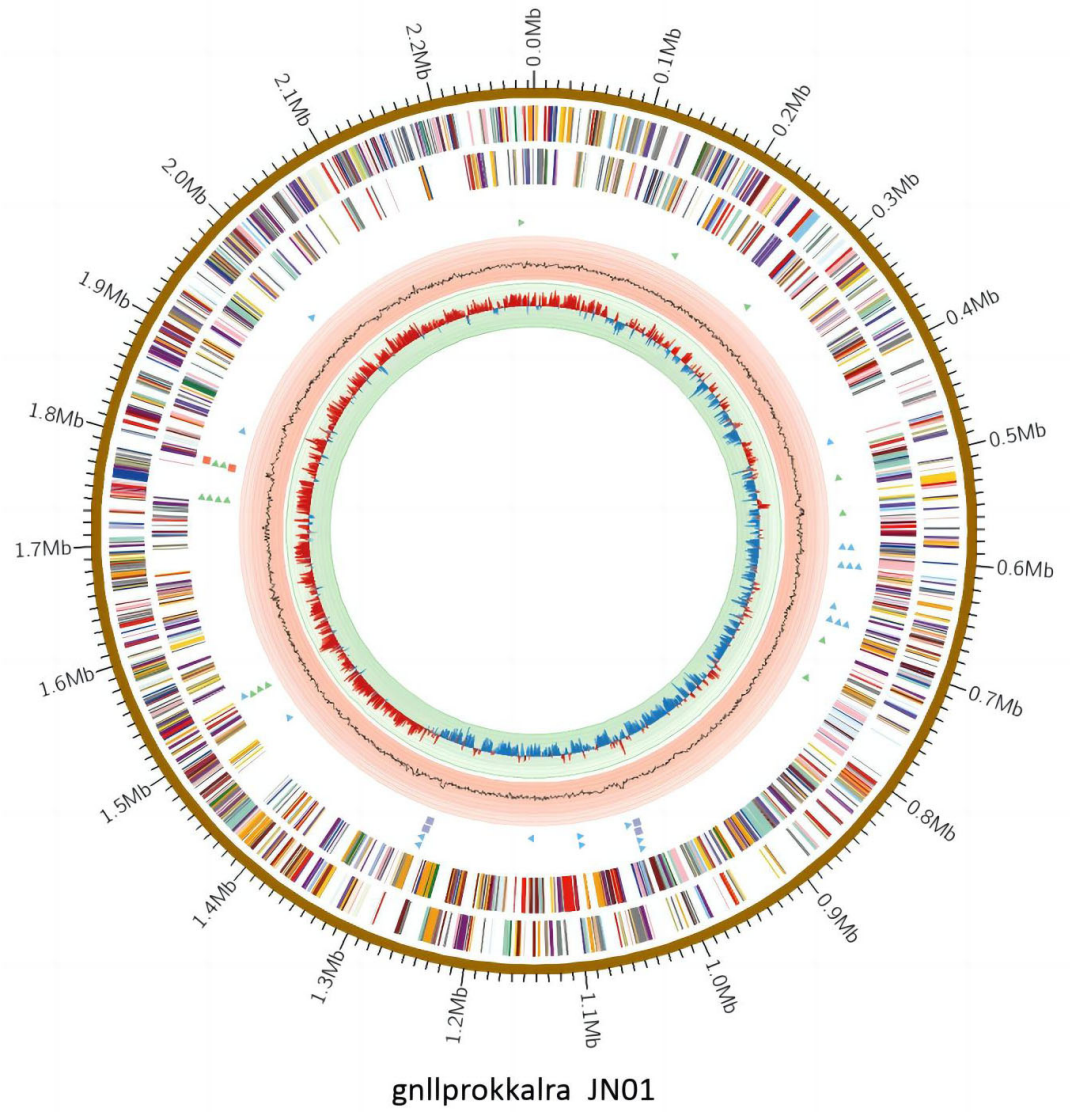
The complete genome of JN01. The outermost circle indicates size of the JN01 genome.

**FIGURE 8:**
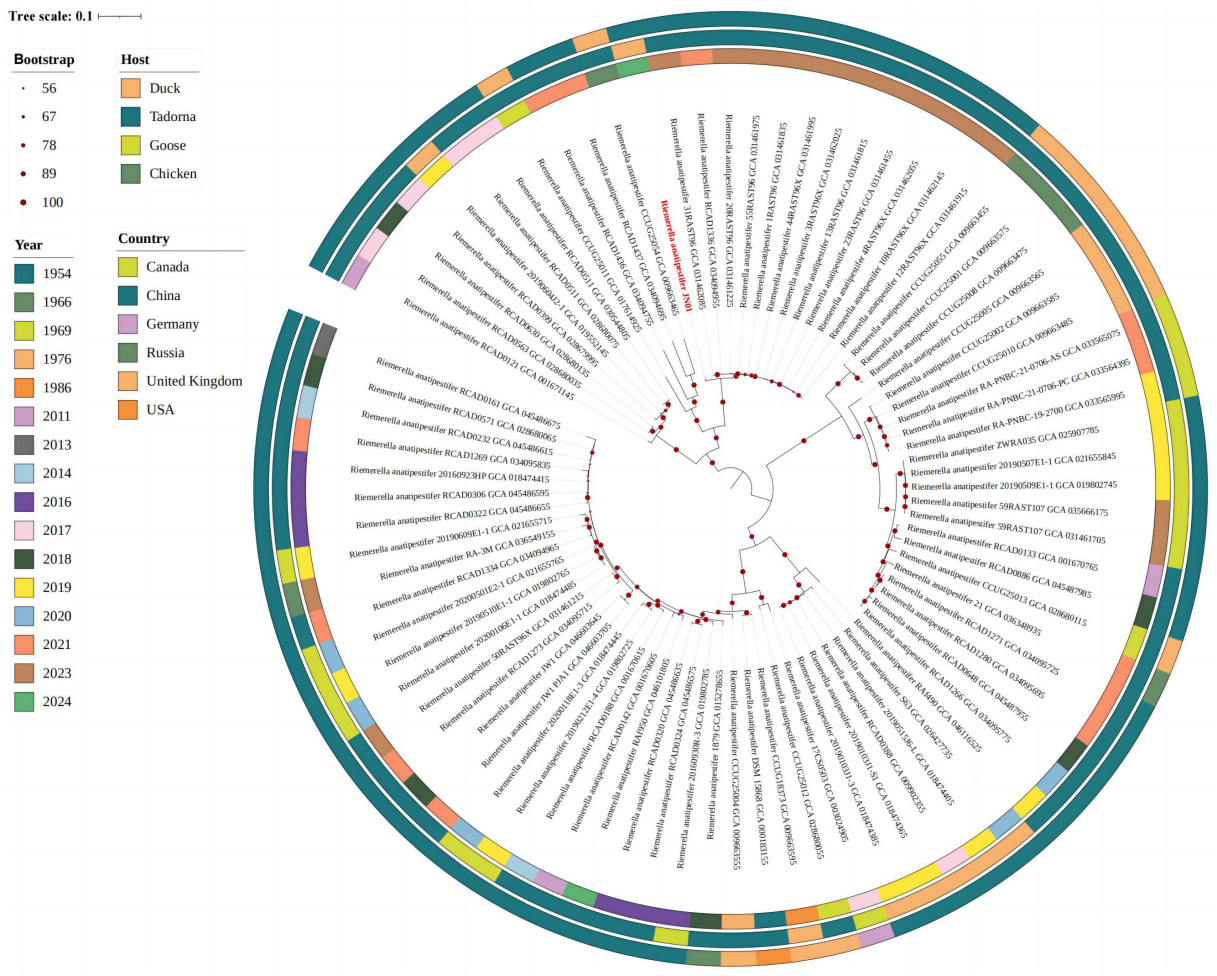
The whole-genome evolutionary tree representing strains from different hosts, eras and countries.

The complete genome of JN01 (serotype 1) comprised 2,284,590 bp with a 34.98% G+C content (GenBank accession no. SAMN47625175), encoding 2,202 predicted genes (including 9 rRNAs and 40 tRNAs) (Figure 7). Three genomic islands were identified, potentially linked to virulence or antibiotic resistance. Phylogenetic analysis (Figure 8) showed JN01 clustered closely with duck-derived strains (e.g., 034094955, 031462085) from China, with no clear host-specific clustering of chicken, duck or goose. The highest diversity of *R. anatipestifer* strains globally was found in China. Meanwhile, this pathogen has also been isolated in other countries such as Asia and the Americas.

## Discussion

This study highlights the emergence of *R. anatipestifer* as a significant pathogen in Chinese chicken flocks in 2023–2024. While historically associated with waterfowl, our findings show its successful host adaptation to chickens. Studies have reported *R. anatipestifer* infections in chickens (Omaleki et al., 2021; Nowaczek et al., 2023), including infection in hens which led to reduced egg productivity, low hatching rates and jelly-like lifeless embryos in 2024 in China (Chen et al., 2024). However, the leg swelling and neurological syndrome occurring frequently in broiler chickens caused by this pathogen have been rarely reported. In this study, both layers and broilers are susceptible to *R. anatipestifer* infection, with the highest isolation rate observed in white-feathered broilers, which is consistent with the findings reported by Zhang et al. (2025). Statistical analysis of bacterial isolation results revealed that *R. anatipestifer* infections predominantly occurred in around 30-day-old broilers, exhibiting the same age-specific susceptibility characteristic as duck (2–4 week old), which suggests conserved pathogenic mechanisms across avian hosts.

The molecular basis for serotyping of *R. anatipestifer* is primarily determined by capsular polysaccharide (CPS) on the surface of the bacterium, as lipopolysaccharide (LPS) only serves as a common antigen (Liu et al., 2023). Notably, the prevalent serotypes exhibit distinct regional characteristics across different geographical areas. In this study, the 28 isolates of chicken-derived *R. anatipestifer* show similar serotypes with slight differences with duck-derived strains (Zhai et al., 2013), where serotypes 1, 2, and 10 predominate. Therefore, the development of multivalent vaccines targeting serotypes 1 and 10 may be effective in controlling local outbreaks of *R. anatipestifer*.

The chicken embryo lethality assay could be used as a first-line screening method to determine the virulence of *R. anatipestifer* strains (Seo et al., 2013). Pathogenicity assays revealed serotype 10 (BZ) as more virulent in chicken embryos. Both serotypes (serotype 1 and serotype 10) induced clinical symptoms in SPF chickens, with preferential tropism for joints and the central nervous system, respectively. Pathology analysis in SPF chickens demonstrated that *R. anatipestifer* infection induced nearly identical clinical signs in chickens and ducks, dominated by neurological signs and dysfunction. Postmortem examination showed characteristic fibrinous deposits across multiple organ systems. The pathogen caused significantly milder respiratory symptoms and lower mortality in chickens compared to ducks, with reported mortality rates in ducks reaching up to 50%, or even 90% (Swayne, 2020). Quantitative detection of *R. anatipestifer* in different tissues demonstrated that the pathogen can penetrate tissue barriers and the blood-brain barrier (BBB) easily, which infiltrates the host’s circulatory and central nervous systems, ultimately causing septicemia and severe neurological dysfunction. Rubbenstroth et al. (2009) found that horizontal transmission was the main mode of *R. anatipestifer* transmission, usually occurring through respiratory tract or skin wounds, particularly skin injuries on the feet. The detection of bacterial DNA in cloacal swabs by 2 dpi highlights early shedding and environmental transmission risks, necessitating strict biosecurity measures.

The whole genome analysis of JN01 can clarify the pathogenic mechanism by dissecting the virulence gene islands, providing targets for the development of targeted drugs and vaccines. The global strain diversity analysis shows that China is a genetic diversity hotspot of *R. anatipestifer*. Combined with the close evolutionary relationship between JN01 and domestic duck-origin strains, continuous monitoring should be strengthened to warn of the emergence and prevalence of new epidemic strains.

Notably, co-infections with *E. coli* and H9 AIV were observed, suggesting synergistic interactions that may exacerbate disease severity. Future studies should investigate host immune responses and co-infection dynamics for prevention and control.

## Data Availability

The datasets presented in this study can be found in online repositories. The names of the repository/repositories and accession number(s) can be found below: https://www.ncbi.nlm.nih.gov/genbank/, SAMN47625175.
